# Genetic and Biochemical Diversity among *Valeriana jatamansi* Populations from Himachal Pradesh

**DOI:** 10.1155/2015/863913

**Published:** 2015-02-08

**Authors:** Sunil Kumar Singh, Rajan Katoch, Rakesh Kumar Kapila

**Affiliations:** ^1^Department of Biotechnology, CSK Himachal Pradesh Krishi Vishvavidyalaya, Palampur 176062, India; ^2^National Research Centre on Plant Biotechnology, Lal Bahadur Shastri Building, Pusa Campus, New Delhi 110012, India

## Abstract

*Valeriana jatamansi* Jones is an important medicinal plant that grows wild in Himachal Pradesh, India. Molecular and biochemical diversity among 13 natural populations from Himachal Pradesh was assessed using RAPD and GC-MS to know the extent of existing variation. A total of seven genetically diverse groups have been identified based on RAPD analysis which corroborated well with the analysis based on chemical constituents. The essential oil yield ranged from 0.6% to 1.66% (v/w). A negative correlation between patchouli alcohol and viridiflorol, the two major valued constituents, limits the scope of their simultaneous improvement. However, other few populations like Chamba-II and Kandi-I were found promising for viridiflorol and patchouli alcohol, respectively. The analysis of chemical constitution of oil of the populations from a specific region revealed predominance of specific constituents indicating possibility of their collection/selection for specific end uses like phytomedicines. The prevalence of genetically diverse groups along with sufficient chemical diversity in a defined region clearly indicates the role of ecology in the maintenance of evolution of this species. Sufficient molecular and biochemical diversity detected among natural populations of this species will form basis for the future improvement.

## 1. Introduction

*Valeriana jatamansi* Jones. (*Valeriana wallichii* DC), also known as Indian valerian, is an erect pubescent herb, having horizontal, thick rootstock/rhizomes, with thick descending fibrous roots [1]. The species is found growing on moist slopes in the Himalayas and Khasi hills in shrubberies and open slopes between 1500 and 4000 m elevation from Pakistan to Southwest China, Burma, and South-East Asia. The plant also grows well in different agroclimatic regions of India. In Himachal Pradesh, it grows profusely in Bharmour division of Chamba, Kanda area of Karsog, and Chansil of Rohru forest division [2].

*Valeriana jatamansi has* long been in use in the Ayurvedic and Unani system of medicine [3]. Herbal medicine remains one of the most common forms of the therapy available for much of the world's population. In traditional medicines, the roots of the plant are used for various ailments like ulcers, convulsions, jaundice, cardiac debility, dry cough, asthma, seminal weakness, chronic and intermittent fevers, skin diseases, falling hairs, nephropathy, leprosy, general debility, and sleep enhancement [4–6]. Besides,* Valeriana jatamansi* is also known to possess fungicidal activity against* Fusarium oxysporum* and* Macrophomina phaseolina* [7].

The medicinal property of the plant is attributed to various chemical components present in its essential oil. The composition of oil has been reported to vary with geographical location and altitude [8–12]. The assessment of the genetic composition of species collected from different phytogeographical regions helps to assess the available diversity, whereas biochemical analysis of economical parts is important to know quantitative as well as qualitative aspects and to choose the economically superior genotypes for the active ingredients.

Among different PCR based markers, random amplified polymorphic DNA (RAPD) is an easy method for discovering random polymorphism in the genome [13]. In contrast to other molecular markers, RAPD is a very simple technique for taxonomic and systemic analysis and phylogenetic studies of plants [14–18]. The usefulness of RAPDs in diversity analysis has been demonstrated at the species level [19], subspecies level [20], population level [21], and cultivar level [22]. The present study was conducted to elucidate the biochemical and molecular diversity among different representative population samples of* Valeriana jatamansi* collected from different geographical locations of Himachal Pradesh, India.

## 2. Materials and Methods

### 2.1. Plant Material

**Figure 1:**
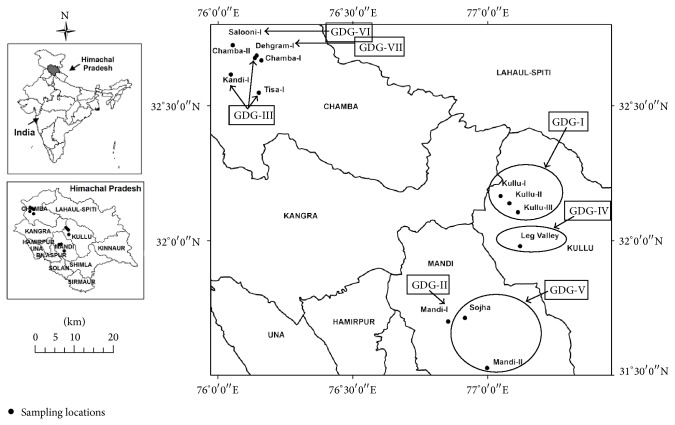
Sampling locations of thirteen populations of* Valeriana jatamansi* from three districts of Himachal Pradesh. The seven genetically diverse groups (GDG) have been indicated by arrow.

A total of 64 samples from thirteen natural populations (five from each population except Mandi-I, having four samples) of* Valeriana jatamansi* Jones (Table 1 and Figure 1) were collected from different location/regions of Himachal Pradesh (HP), India, for the present study. Since the collections were made from the available natural populations at different locations, representative samples collected were limited in number ranging from 4 to 5.

### 2.2. DNA Extraction and PCR Amplification

Genomic DNA was isolated from young leaves using CTAB method given by Murray and Thompson [23]. The isolated genomic DNA was stored at −20°C until being used. Decamer primers of arbitrary sequence from operon (A, C, D, E, F, J, P, Q, and X series) were used to amplify genomic DNA of twenty-six samples (2 per collections) and based on polymorphism obtained forty-five primers were selected (Table 2) for final amplification of all the collections. DNA amplification was carried out by making final reaction volume of 20 *μ*L containing 1.6 *μ*L of dNTP mix (0.2 mM each of dATP, dGTP, dCTP, and dTTP), 0.16 *μ*L of* Taq* DNA polymerase (5 U/*μ*L), 2.0 *μ*L DNA template (25 ng/*μ*L), 1.0 *μ*L of 5 *μ*M primer, 2.0 *μ*L of 10X PCR buffer, 1.2 *μ*L of MgCl_2_ (25 mM), and 12.04 *μ*L of sterilized distilled water.

The DNA amplification was carried out in a thermal cycler (Applied Biosystems). The PCR program was set at initial cycle of 94°C for 5 minutes, 37°C for 1 min, and 72°C for 2 min. Further amplification was repeated 40 times consisting of denaturation at 94°C for 1 minute, annealing at 37°C for 1 min and extension at 72°C for 2 min. Final extension of 5 min at 72°C was carried out before rapid cooling to 4°C. Amplification products were separated by agarose gel electrophoresis in 1 X TAE on 1.4% agarose containing 0.5 ng/*μ*L ethidium bromide. Images were photographed and captured by Gel Doc (Bio-Rad). Molecular weights were estimated using a 1000 bp DNA ladder.

### 2.3. RAPD Data Scoring and Analysis

The RAPD profiles generated by different primers were compared to determine relatedness within and among different populations. The presence and absence of each RAPD band of a particular molecular weight in all genotypes were scored manually. A binary data matrix with “1” indicating presence of a particular molecular weight band and “0” indicating its absence was generated separately for each primer. The binary data were used for principal coordinate analysis (PCA), analysis of molecular variance (AMOVA), and mantel test [24] of geographic and genetic distance using GenAlEx software [25]. PCA was done based on genetic distance measure calculated from binary data for multiple samples with multiple populations using GenAlEx software. The neighbor-joining tree and bootstrap analysis were executed using DARWIN version 6.0 [26]. The statistical analysis was done by using StatSoft Inc. [27], STATISTICA (data analysis software system), version 7. Fst and Nm values were calculated using GenAlEx software following formula given by Nei [28, 29].

### 2.4. GC-MS Analysis of Essential Oil

Roots and rhizomes of* Valeriana jatamansi* dried for 20 days under ambient room conditions in shade [30, 31] were used for essential oil extraction by hydrodistillation in Clevenger apparatus. The essential oil was dried over anhydrous Na_2_SO_4_. The purified fraction was used for recording GC-MS data. Two *μ*L of essential oil fraction was used for injection. GC-MS (70 eV) data were measured in MS-QP-2010 series Shimadzu, Tokyo, Japan, equipped with MS, AOC-20i autosampler, and BP-20 capillary column (SGC International, Ringwood, Australia) 30 m length, 0.25 mm I.D., and film thickness 0.25 *μ*m (poly ethylene glycol), with helium as a carrier gas. The injector temperature was 220°C with split ratio of 1 : 50. The GC oven temperature was programmed to hold at 70°C for 4 min and then to increase up to 220°C at increments of 4°C/min and finally it holds at 220°C for 5 min. Column flow rate was set at 1.10 mL/min. Ion source temperature was 200°C and interface temperature was set at 220°C. The MS was scanned at 70 eV over 40–600 a.m.u. The individual components of the essential oils were identified by comparing their mass spectra with a computerized MS-database using WILEY7, NIST 147, NIST 27, and SZTERP libraries.

## 3. Results and Discussion

### 3.1. RAPD Polymorphism

**Figure 2:**
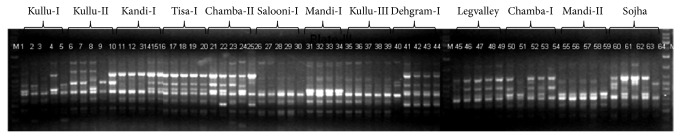
RAPD profile of thirteen populations of* Valeriana jatamansi*.

A total of 150 primers were initially screened for amplification of DNA of a subset of 26 samples (two plants randomly selected from each population). Based on the polymorphic information content (PIC) [32], the signal intensity, and number of bands, 45 primers were selected for final analysis (Table 2). The representative RAPD profile generated by OPA-3 primer is shown in Figure 2. All forty-five primers, generated a total of 368 bands with a mean of 8 bands per primer ranging from 3 to 15 per primer. Of 368 bands, only 75 (20.39%) amplified fragments were present in all the 64 plants, whereas 293 (79.61%) were polymorphic. It indicated considerable variation among the 64 samples of 13 populations. Kumar [33] had reported 90.18%, while Rajkumar et al. [34] reported a range of 65–81% polymorphism in the sampled populations of the species in their studies. The difference in the level of polymorphism among these reports might be due to less number of primers used by Kumar [33] and lower number of polymorphic loci (241) obtained by Rajkumar et al. [34], as compared to the present investigation. Besides, it can also be due to inherent differences in the samples collected from different geographical regions in our case.

### 3.2. Genetic Diversity Analysis and Population Structure

**Figure 3:**
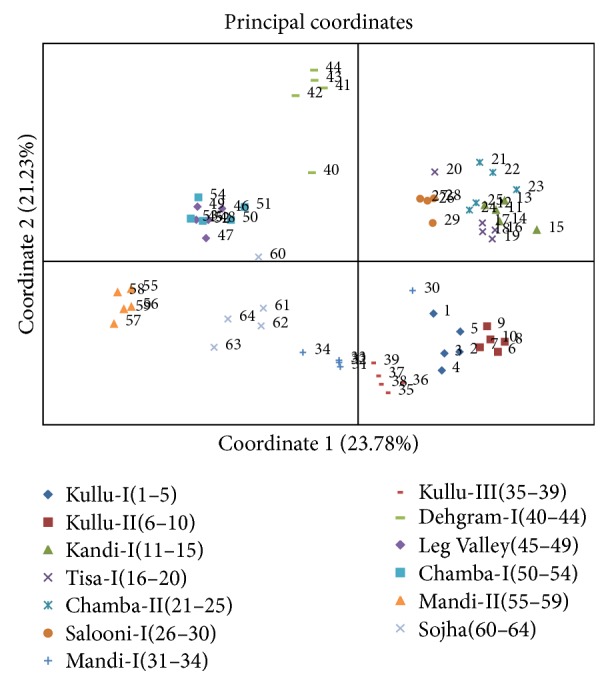
Principal coordinate analysis of genetic differences among 13 populations of* Valeriana jatamansi*. Values in parenthesis show level of variation explained by the coordinate.

**Figure 4:**
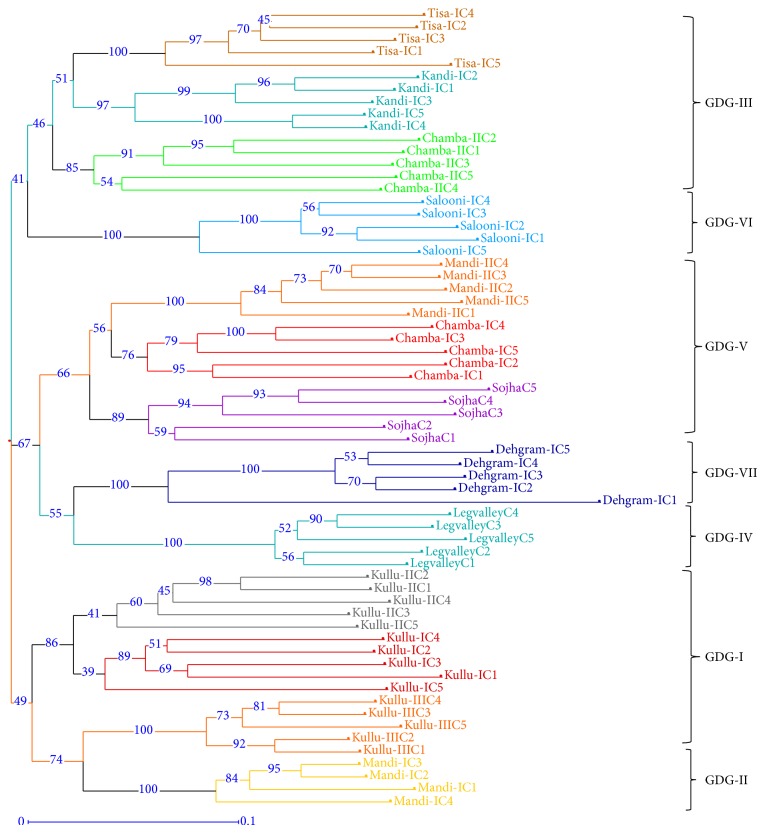
Dendrogram of 64 samples from 13 populations of* Valeriana jatamansi *representing clustering of samples in a separate group showing the population specific grouping. Cluster analysis was performed using the neighbor-joining method. Bootstrap values obtained from 500 replicate analyses higher than 40% are indicated on nodes.

**Figure 5:**
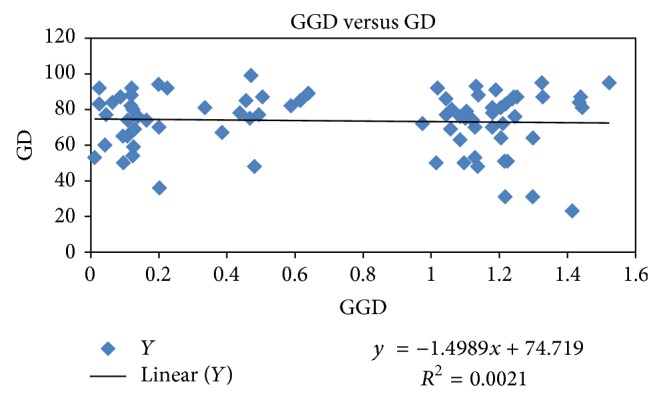
Test of correlation between genetic and geographic distances among 13 populations of* Valeriana jatamansi*.

The binary data used for principal coordinates analysis (PCA) distributed the samples in two coordinates; coordinate one accounted for 23.78%, whereas coordinate two accounted for 21.23% of the total variation among populations (Figure 3). Distribution pattern of all the samples from different populations revealed consistency with their geographical origin. It clearly revealed lesser intrapopulation variation as compared to interpopulation variation. The same was evident from the analysis of molecular variance (AMOVA) wherein 48% (*P* = 0.001) variation was recorded within population as compared to 52% (*P* = 0.001) among populations (Table 3). The pairwise differences (Fst) between populations (calculated based on allele sharing) varied from 0.25 to 0.74 (Table 4). The estimated extent of gene flow (Nm) among populations is 0.253, ranging from 0.08 (between populations from Salooni-I and Leg Valley-II) to 0.72 (between populations from Kullu-I and Kullu-II). Based on Nei's genetic similarity index (Table 4), 13 populations clustered in seven different groups are designated hereafter as genetically diverse groups (GDGs) at 85% genetic similarity level (Figure 4, Table 4). This clustering into seven GDGs was further corroborated based on biochemical profiling of their essential oil. As evident from Figures 1, 3, and 4, most of the samples from a particular area/region (population) were grouped separately, deciphering the level and robustness of diversity analysis using large number of RAPD markers with high PIC value encompassing larger genome coverage. The populations from specific regions such as Kullu, Chamba, and Mandi are grouped together except some of the samples that exhibited variation and were grouped in different clusters. This possibly can be due to their natural habitat and geographic confinement. The clustering pattern further indicates that the populations are not much differentiated during the evolution and the slight genetic variation present within population(s) might have evolved under the influence of environmental factors. The mantel test did not exhibit significant correlation (*r* = 0.002; *P* = 0.05) between genetic distance and geographic distance of populations (Figure 5).

### 3.3. Essential Oil Composition and Chemical Diversity

The essential oil yield from roots was found to vary from 0.6% to 1.66% (Table 5) with a mean oil yield of 1.090 ± 0.052 among 13 populations studied. The DMRT analysis revealed that the populations differ in oil content significantly from each other with Tisa-I and Dehgram-I having highest oil content as compared to others, while populations Leg valley, Chamba-I, Mandi-II, and Kullu-I have the lowest oil content. Based on the GC and GCMS analysis, ten major chemical constituents were identified in all the populations, namely, endobornyl acetate (0 to 4.56%), calarene (0.58 to 24.21%),* alpha*-guaiene (0.51 to 4.16%), seychellene (0.89 to 5.29%), azulene (0.24 to 6.73%), selinene (0.38 to 2.21%), viridiflorol (1.82 to 48.8%), epiglobulol (0 to 3.16%), patchouli alcohol (0.98 to 65.04%), and pogostol (0 to 2.39%). The correlation analysis indicated a negative trend of major chemical constituents and essential oil with altitude, though it was not significant (Table 6), while a significant positive and negative correlation exists among the chemical constituents of essential oil, for example, calarene has a significant negative trend in relation to patchouli alcohol, seychellene, and pogostol, whereas with epiglobulol it is positive. This indicates that the accumulation of one type of constituent hinders the accumulation of other forms. The variation in chemical composition of essential oil with change of altitude has already been documented in the literature [9, 35]. Amongst constituents, patchouli alcohol content was found to have significantly high positive correlation with* alpha*-guaiene (0.816), seychellene (0.884), and azulene (0.602), while it has significant negative association with calarene (−0.953), viridiflorol (−0.820), and epiglobulol (−0.954). As the patchouli alcohol and viridiflorol are the major economic constituents of* Valeriana jatamansi*, these correlation analyses will provide a yardstick for the selection of plants from the population having high contents of desired constituent in the essential oil. It is also evident from the analysis that the two major chemical constituents are negatively correlated and selection for one will hinder the selection of the other. Hence, for a particular type of chemical constituent and end use, one has to choose specific population. In present study, Chamba-II and Kandi-I were observed as best populations for viridiflorol and patchouli alcohol, respectively.

Singh et al. [36] reported prevalence of chemotypes of* Valeriana jatamansi* in Himachal Pradesh according to the area of their natural habitat.  Table 7 documents the area wise diversity of genetic groups present and the chemical diversity documented for each group in a particular area. The table clearly indicates that there is prevalence of microclimate on the chemical composition of the essential oil from plants inhabiting the particular area. For example, the samples from Kullu region have been divided into two distinct groups on the basis of genetic identity and the chemical constituents also corroborating molecular analysis. Based upon information generated under the present investigation, specific population of a region can be selected and targeted for a particular chemical constituent; for example, the samples collected from Chamba region will in general be having highest level of oil content (1.48%) with highest chemical constituents such as viridiflorol (22.31%), longifolenaldehyde (2.02%), calarene (9.18%), and* alpha*-patchoulene (4.98%). Similarly samples from Mandi could be targeted for highest level of pogostol (2.10%), (−)-*alpha*-selinene (2.33%), and spathulenol (0.95%).

## 4. Conclusion

RAPD profiling of 13 populations of* Valeriana jatamansi* with 45 oligo primers generated a total of 368 amplicons and showed 79.61% polymorphism as a whole. The PCA and AMOVA revealed that variation among populations was slightly higher than that within population(s). It might be due to local environmental effect during adaptation of plant to given environment. The chemical analysis of essential oil, which ranged from 0.6% to 1.66% (v/w), led to identification of ten major chemical constituents, namely, endobornyl acetate (0 to 4.56%), calarene (0.58 to 24.21%),* alpha*-guaiene (0.51 to 4.16%), seychellene (0.89 to 5.29%), azulene (0.24 to 6.73%), selinene (0.38 to 2.21%), viridiflorol (1.82 to 48.8%), epiglobulol (0 to 3.16%), patchouli alcohol (0.98 to 65.04%), and pogostol (0 to 2.39%). Two major components of oils, namely, patchouli alcohol and viridiflorol, exhibited negative association, thus limiting scope of simultaneous gains from selection for both constituents. Chamba-II and Kandi-I populations were found to be best populations for viridiflorol and patchouli alcohol, respectively. Since variable contents of patchouli alcohol and viridiflorol can potentially affect therapeutic efficacy of the plant, it will be important to understand and consider negative association between two major constituents while making any attempts of genetic enhancement in order to breed new cultivars with more desirable chemical constituents. This study also provides indicative guideline for collection of samples from a suitable region for a particular end use based on the predominance of particular chemical constituents in its oil.

## Figures and Tables

**Table 1:** Geographical descriptors of collections of *Valeriana jatamansi* from Himachal Pradesh, India, used in the present study.

Population	Sample size	Altitude (m)	Latitude	Longitude
Kullu-I	5	3647	32°09′56′′N	77°02′41′′E
Kullu-II	5	2734	32°08′19′′N	77°04′37′′E
Kandi-I	5	854	32°36′58′′N	76°02′42′′E
Tisa-I	5	1220	32°32′54′′N	76°08′55′′E
Chamba-II	5	2104	32°40′36′′N	76°08′03′′E
Salooni-I	5	1730	32°43′29′′N	76°03′08′′E
Mandi-I	4	764	31°42′00′′N	76°51′00′′E
Kullu-III	5	2541	32°06′20′′N	77°06′30′′E
Dehgram-I	5	2165	32°41′11′′N	76°08′25′′E
Leg Valley	5	1720	31°58′47′′N	77°06′59′′E
Chamba-I	5	2368	32°40′06′′N	76°09′29′′E
Mandi-II	5	945	31°31′41′′N	76°59′41′′E
Sojha	5	2692	31°42′47′′N	76°54′47′′E

**Table 2:** Level of polymorphism detected using 45 RAPD primers in 13 populations of *Valeriana jatamansi*.

Marker	Scored bands	Polymorphic bands	PIC value (%)
OPA-01	9	8	85.40
OPA-02	9	9	83.29
OPA-03	9	9	87.88
OPA-04	10	9	83.82
OPA-09	7	6	81.93
OPA-11	11	10	88.58
OPA-13	10	4	87.75
OPA-14	10	7	85.70
OPA-15	8	6	84.19
OPA-19	9	8	86.78
OPA-20	10	7	82.40
OPC-06	11	11	89.23
OPC-20	6	5	67.19
OPD-07	7	5	84.03
OPD-12	9	9	82.70
OPD-13	9	9	85.32
OPD-16	6	5	79.51
OPD-18	5	5	76.81
OPD-19	7	7	77.39
OPE-07	7	6	79.18
OPF-01	3	2	63.95
OPF-02	6	5	79.31
OPF-10	7	7	84.44
OPF-12	7	5	66.44
OPF-13	12	12	88.06
OPJ-01	3	2	55.40
OPJ-04	11	10	85.73
OPJ-10	10	10	85.62
OPJ-11	5	4	75.72
OPJ-14	15	14	92.25
OPJ-18	7	5	81.57
OPP-08	10	8	86.76
OPP-10	7	2	84.93
OPP-11	8	4	86.01
OPQ-01	6	5	86.08
OPQ-04	12	7	88.57
OPQ-06	8	7	82.25
OPQ-09	8	1	80.22
OPQ-12	9	8	83.28
OPQ-13	8	8	82.25
OPQ-14	4	1	70.09
OPQ-15	6	3	77.15
OPQ-16	12	6	89.59
OPX-02	8	6	81.86
OPX-19	7	6	84.20

Total	368	293	81.79 (mean)

**Table 3:** Analysis of molecular variance (AMOVA) of 64 samples of 13 populations of *Valeriana jatamansi*.

Source	df	Sum of squares	Mean squared deviation	Estimated variance	% Total variance	Probability
Among populations	12	1778.137	148.178	25.337%	52%	<0.001
Within populations	51	1197.050	23.472	23.472%	48%	<0.001

df = degree of freedom, SS = sum of squares, and MS = mean square.

**Table 4:** Pairwise Fst and Nm (in parenthesis) value among populations of *Valeriana jatamansi* (below diagonal) and and Nei's genetic similarity (above diagonal) index.

	Kullu-I	Kullu-II	Kandi-I	Tisa-I	Chamba-II	Salooni-I	Mandi-I	Kullu-III	Dehgram-I	Leg Valley	Chamba-I	Mandi-II	Sojha
Kullu-I		0.903	0.853	0.828	0.838	0.805	0.846	0.858	0.784	0.812	0.812	0.801	0.842

Kullu-II	0.256 (0.727)		0.869	0.822	0.839	0.780	0.825	0.853	0.770	0.798	0.806	0.778	0.822

Kandi-I	0.407 (0.364)	0.394 (0.384)		0.873	0.872	0.823	0.789	0.807	0.812	0.793	0.837	0.773	0.831

Tisa-I	0.467 (0.285)	0.501 (0.249)	0.421 (0.344)		0.875	0.790	0.799	0.805	0.782	0.799	0.820	0.784	0.806

Chamba-II	0.374 (0.418)	0.393 (0.385)	0.342 (0.481)	0.347 (0.471)		0.847	0.808	0.821	0.829	0.799	0.835	0.778	0.806

Salooni-I	0.528 (0.193)	0.586 (0.152)	0.549 (0.173)	0.609 (0.137)	0.448 (0.308)		0.811	0.801	0.770	0.743	0.825	0.787	0.785

Mandi-I	0.453 (0.419)	0.521 (0.314)	0.603 (0.244)	0.605 (0.230)	0.419 (0.347)	0.529 (0.223)		0.851	0.750	0.812	0.812	0.837	0.801

Kullu-III	0.451 (0.304)	0.488 (0.262)	0.593 (0.172)	0.609 (0.160)	0.498 (0.252)	0.698 (0.108)	0.468 (0.284)		0.773	0.785	0.812	0.813	0.837

Dehgram-I	0.525 (0.226)	0.565 (0.193)	0.529 (0.223)	0.583 (0.179)	0.433 (0.327)	0.648 (0.136)	0.575 (0.185)	0.636 (0.143)		0.820	0.830	0.776	0.777

Leg Valley	0.533 (0.219)	0.579 (0.182)	0.609 (0.161)	0.615 (0.157)	0.530 (0.222)	0.740 (0.088)	0.562 (0.195)	0.680 (0.118)	0.572 (0.187)		0.855	0.829	0.813

Chamba-I	0.468 (0.284)	0.499 (0.251)	0.469 (0.283)	0.510 (0.240)	0.407 (0.364)	0.562 (0.195)	0.463 (0.289)	0.567 (0.191)	0.483 (0.268)	0.490 (0.261)		0.881	0.881

Mandi-II	0.550 (0.205)	0.604 (0.164)	0.633 (0.145)	0.633 (0.145)	0.558 (0.198)	0.706 (0.104)	0.501 (0.249)	0.644 (0.138)	0.630 (0.147)	0.619 (0.154)	0.427 (0.335)		0.872

Sojha	0.390 (0.391)	0.450 (0.306)	0.454 (0.301)	0.507 (0.243)	0.434 (0.327)	0.596 (0.170)	0.458 (0.296)	0.498 (0.252)	0.538 (0.215)	0.537 (0.215)	0.329 (0.510)	0.422 (0.342)	

**Table 5:** Essential oil contents and their major constituents in 13 populations of *Valeriana jatamansi*.

	Endobornyl acetate (%)	Calarene (%)	*alpha*-Guaiene (%)	Seychellene (%)	Azulene (%)	Selinene (%)	Viridiflorol (%)	Epiglobulol (%)	Patchouli alcohol (%)	Pogostol (%)	Oil (DW) (Vol/Wt) %
Kullu-I	2.1	2.04	2.15	2.97	2.49	1.17	16.62	0.25	56.29	2.1	0.67^h^
Kullu-II	4.56	15.43	0.55	2.27	1.33	1.22	29.1	1.67	14.73	0	1.17^e^
Kandi-I	0.84	0.58	3.54	4.39	6.23	2.12	1.82	0.0	65.04	2.36	1.37^cd^
Tisa-I	0.0	24.21	0.73	0.89	0.51	1.99	26.72	3.16	0.98	0.0	1.66^a^
Chamba-II	2.72	10.41	0.51	1.39	0.4	0.38	48.8	1.49	15.7	0.75	1.12^ef^
Salooni-I	0.76	2.35	1.23	3.77	1.53	0.85	14.92	0.26	59.29	2.29	1.5^bc^
Mandi-I	0.27	3.09	2.6	3.46	3.75	1.43	19.58	0.34	50.96	2.15	0.93^g^
Kullu-III	0.5	2.39	2.63	3.56	5.15	1.57	15.53	0.21	48.47	2.12	1.25^de^
Dehgram-I	1.99	13.46	1.19	2.43	1.58	0.93	22.71	1.7	30.16	1.32	1.56^ab^
Leg Valley	0.0	0.72	4.16	5.29	6.73	2.12	3.15	0.0	60.17	2.27	0.64^h^
Chamba-I	0.26	1.55	3.04	3.12	0.24	1.95	6.42	0.0	59.93	2.39	0.69^h^
Mandi-II	0.76	7.06	2.42	3.13	3.89	1.67	24.47	0.76	38.52	1.66	0.6^h^
Sojha	1.01	1.07	3.18	3.94	5.84	2.21	8.07	0.0	59.11	2.11	1.0^fg^

Superscripts on oil content value denote significant homogeneous grouping at *P* ≤ 0.05 using DMRT.

**Table 6:** Correlation among essential oil contents, its constituents, and altitude of growth habitat in 13 *Valeriana jatamansi* populations.

	Endobornyl acetate	Calarene	*alpha*-Guaiene	Seychellene	Azulene	Selinene	Viridiflorol	Epiglobulol	Patchouli alcohol	Pogostol	Oil (DW)	Altitude
Endobornyl acetate	1	0.334	−0.598^*^	−0.407	−0.37	−0.584^*^	0.554^*^	0.301	−0.445	−0.558^*^	0.091	0.502
Calarene		1	−0.774^**^	−0.844^**^	−0.600^*^	−0.224	0.649^*^	0.993^**^	−0.953^**^	−0.940^**^	0.535	−0.102
*alpha*-Guaiene			1	0.862^**^	0.804^**^	0.728^**^	−0.837^**^	−0.785^**^	0.816^**^	0.796^**^	−0.358	−0.148
Seychellene				1	0.816^**^	0.469	−0.819^**^	−0.861^**^	0.884^**^	0.824^**^	−0.366	−0.105
Azulene					1	0.584^*^	−0.625^*^	−0.606^*^	0.602^*^	0.561^*^	−0.271	−0.194
Selinene						1	−0.734^**^	−0.265	0.362	0.295	−0.229	−0.244
Viridiflorol							1	0.680^*^	−0.820^**^	−0.742^**^	0.289	0.042
Epiglobulol								1	−0.954^**^	−0.924^**^	0.432	−0.118
Patchouli alcohol									1	0.969^**^	−0.387	0.045
pogostol										1	−0.417	−0.03
Oil (DW)											1	−0.186
Altitude												1

^*^Significant at 0.05 level. ^**^Significant at 0.01 level.

**Table 7:** Highly diverse biochemical profile revealed by oil constituents analysis of population from different ecological niches which also possess high level of genetic diversity.

GDG	Kullu			Mandi			Chamba			
	I	IV	Avg.	V	II	Avg.	III	VI	VII	Avg.
Viridiflorol (%)	21.13	3.15	**12.14**	13.24	19.92	**16.58**	27.33	15.18	24.41	**22.31**
Isopatchoulane (%)	0.62	0.67	**0.64**	0.60	0.55	**0.57**	0.81	0.87	0.45	**0.71**
Longifolenaldehyde (%)	0.91	1.53	**1.22**	1.86	0.73	**1.30**	3.80	0.38	1.89	**2.02**
Patchouli alcohol (%)	39.83	60.17	**50.00**	52.52	50.96	**51.74**	27.24	59.29	30.16	**38.90**
Pogostol (%)	1.41	2.27	**1.84**	2.05	2.15	**2.10**	1.04	2.29	1.32	**1.55**
Juniper camphor (%)	1.55	1.55	**1.55**	1.49	1.66	**1.58**	2.10	1.14	0.81	**1.35**
DL-Limonene (%)	0.42	0.34	**0.38**	0.33	0.24	**0.28**	0.29	0.00	0.00	**0.10**
Camphene (%)	0.90	0.50	**0.70**	0.41	0.22	**0.32**	0.25	0.00	0.00	**0.08**
*p*-Cymene (%)	0.93	0.00	**0.47**	0.17	0.00	**0.08**	0.42	0.00	0.00	**0.14**
*beta*-Patchoulene (%)	1.37	1.32	**1.35**	1.03	0.64	**0.84**	0.11	0.00	0.00	**0.04**
Endobornyl Acetate (%)	2.39	0.00	**1.19**	0.68	0.27	**0.47**	1.19	0.76	1.99	**1.31**
Calarene (%)	6.62	0.72	**3.67**	3.23	3.09	**3.16**	11.73	2.35	13.46	**9.18**
*alpha*-Guaiene (%)	1.78	4.16	**2.97**	2.88	2.60	**2.74**	1.59	1.23	1.19	**1.34**
*cis*-Farnesol (%)	0.13	0.00	**0.06**	0.26	0.46	**0.36**	0.20	2.43	0.34	**0.99**
*alpha*-Patchoulene (%)	0.89	0.58	**0.74**	2.12	0.00	**1.06**	5.65	1.07	8.22	**4.98**
Seychellene (%)	2.93	5.29	**4.11**	3.40	3.46	**3.43**	2.22	3.77	2.43	**2.81**
*alpha*-Humulene (%)	0.99	1.86	**1.43**	1.52	1.14	**1.33**	1.06	0.70	0.83	**0.86**
Azulene (%)	2.99	6.73	**4.86**	3.32	3.75	**3.54**	2.38	1.53	1.58	**1.83**
(−)-*alpha*-Selinene (%)	1.34	3.07	**2.20**	2.60	2.06	**2.33**	2.74	0.85	0.93	**1.51**
*ar*-Curcumene (%)	0.44	0.00	**0.22**	0.29	0.00	**0.15**	0.54	0.00	0.24	**0.26**
Isoaromadendrene epoxide (%)	0.15	0.37	**0.26**	0.06	0.42	**0.24**	0.27	0.78	0.51	**0.52**
Valerenal (%)	0.53	0.47	**0.50**	0.14	0.80	**0.47**	0.74	0.26	0.00	**0.33**
Spathulenol (%)	0.60	0.94	**0.77**	0.44	1.45	**0.95**	0.14	0.24	0.00	**0.13**
Oil content (%)	1.03	0.64	**0.84**	0.76	0.93	**0.85**	1.38	1.50	1.56	**1.48**
